# ^99m^Tc-labelled Stealth® liposomal doxorubicin (Caelyx®) in glioblastomas and metastatic brain tumours

**DOI:** 10.1038/sj.bjc.6600093

**Published:** 2002-02-12

**Authors:** P Laverman, O C Boerman, G Storm, W J G Oyen

**Affiliations:** Department of Nuclear Medicine, University Medical Center Nijmegen, Nijmegen, The Netherlands; Department of Pharmaceutics, Utrecht University, Utrecht, The Netherlands

## Abstract

*British Journal of Cancer* (2002) **86**, 659–660. DOI: 10.1038/sj/bjc/6600093 www.bjcancer.com

© 2002 Cancer Research UK

## Sir

We read with interest the article by Koukourakis *et al* (2000a) describing the use of radiolabelled Caelyx® to study the accumulation of the polyethylene glycol (PEG)-liposomes in glioblastomas and metastatic brain tumours, and would like to take the opportunity to make some principal remarks regarding the applied radiolabelling procedure.

To facilitate imaging of the accumulation of the liposomes in the tumours, the authors labelled the liposomal formulation with technetium-99m-diethylenetriamine pentaacetic acid (^99m^Tc-DTPA). This labelling method was described previously in more detail by the same group (Koukourakis *et al*, 1999). However, we have some major concerns about the labelling method and imaging protocol used in their studies (Koukourakis *et al*, 1999, 2000a,b).

The applied labelling method is based on the assumption that adding ^99m^Tc-DTPA to the PEG-liposomes (in the Caelyx® formulation) results in radiolabelled liposomes. This method, which has not been described in the literature before, is expected to yield an unstable radiolabelled product, since there is no driving force that will facilitate the hydrophilic ^99m^Tc-DTPA to pass the lipid bilayer and entrap the radiolabel inside the liposome. Moreover, the described quality control method (Koukourakis *et al*, 1999, 2000a,b) does not discriminate between free ^99m^Tc-DTPA and liposome-associated ^99m^Tc-activity. The chromatographic method applied can distinguish between reduced ^99m^TcO_2_ and unreduced ^99m^TcO_4_^-^, however, it is more essential to discriminate between liposome-associated ^99m^Tc-activity and ^99m^Tc-DTPA in the preparation. The proper way to examine this, would be to elute a sample of the radiolabelled liposomes on a gelpermeation column as described previously (Dams *et al*, 2000; Harrington *et al*, 2001). The (radiolabelled) liposomes will elute with the void volume in the early fractions, whereas the non-liposome-associated radiolabel (both as ^99m^Tc-DTPA, ^99m^TcO_2_, and ^99m^TcO_4_^-^) will elute in later fractions.

**Figure 1:**
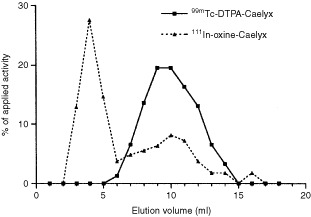
Elution profile of ^99m^Tc-DTPA-Caelyx® (labelled according to Koukourakis *et al*, 1999) and ^111^In-oxinate-Caelyx® (labelled according to Laverman *et al*, 2001) on a 10 ml Sephadex G25 column. A sample of 100 μl was applied. Twenty consecutive fractions of 1 ml were eluted with 5% glucose and the activity of each fraction was counted in a gamma-counter. Note that the radiolabelled liposomes elute in fractions 3–5, whereas the free radiolabel elutes in fractions 6–14.

Our attempts to reproduce the labelling method as described by Koukourakis *et al* (2000a) failed. Analysis of the labelling mixture by instant thin layer chromatography (ITLC) according to Koukourakis *et al* (2000a) showed the same results as described in their article, suggesting a labelling efficiency of approximately 80%. However, analysis on a Sephadex G25-column revealed that only 1% of the ^99m^Tc activity was associated with the liposomes and the majority of the activity eluted in later fractions ( Figure 1

). This was confirmed by ITLC on silica strips in sodium citrate, a method to distinguish ^99m^Tc-DTPA and ^99m^Tc-liposomes described previously (Cao and Suresh, 2000).

Our second point of concern is the chosen time-point of imaging. The patients were imaged at 2 h after infusion of the liposomes (Koukourakis *et al*, 2000a). Harrington *et al* (2001) showed that clear visualization of solid tumours with ^111^In-labelled long-circulating PEG-liposomes was achieved not earlier than 48–72 h after injection, due to the high blood background signal at earlier time-points. These findings are in line with our clinical study in patients with infectious or inflammatory disease, imaged with similar ^99m^Tc-PEG-liposomes, labelled with ^99m^Tc-hexamethylpropylene-amine-oxime (Dams *et al*, 2000). Therefore, it appears that the images showing liposome uptake in the tumours (Koukourakis *et al*, 2000a) represent the accumulation of ^99m^Tc-DTPA, rather than uptake of the radiolabelled Caelyx. ^99m^Tc-DTPA was used in the past for diagnosis of a disrupted blood–brain barrier in brain tumours, before the CT and MRI era (Hauser *et al*, 1970). Nowadays, ^99m^Tc-DTPA is a well-known agent for evaluation of renal function. It is cleared rapidly and efficiently from the circulation by glomerular filtration. This might explain, for liposomes, the unusual, rapid accumulation of activity in the tumours. In our opinion, the authors should have performed, and shown – at least in some patients – a control ^99m^Tc-DTPA scan, to rule out that the liposome scan represents free ^99m^Tc-DTPA instead of radiolabelled Caelyx®.

A better approach to label Caelyx® would be the labelling with indium-111-oxine (^111^In-oxine). This easy method will yield radiolabelled liposomes with good radiochemical yield (>80%) and good *in vivo* stability (Laverman *et al*, 2001). An additional advantage is the longer physical half-life of ^111^In, which enables the acquisition of delayed images and thus better visualization of the tumours (Harrington *et al*, 2001).

In summary, scintigraphic techniques are very helpful in investigating the *in vivo* distribution of (new) pharmaceuticals, but should only be performed using well-established labelling techniques and quality control methods. The results presented by Koukourakis *et al* (1999, 2000a,b) should therefore be interpreted with caution.
